# Medical Opinion and Movement

**Published:** 1911-09-23

**Authors:** 


### Medical Opinion and Movement.

### Death from Convulsions.

At a recent inquest a medical man was blamed for not ascertaining at the time of his first visit the cause of convulsions in a child. The medical man was annoyed at being blamed for carelessness or ignorance, and not without reason, while the coroner apparently had no sympathy with the attitude adopted by the doctor towards the opinion of the jury. Not only is it extremely difficult, or even impossible, to ascertain the nature of convulsions occurring in an infant during life, but after death the cause may still remain obscure. Occasionally an infant, to all appearances in every way healthy, may be seen on the post-mortem table after having succumbed to repeated convulsions. Careful investigation fails to reveal any cause for the convulsions. All the organs are healthy, and no probable focus for the production of toxins, such as pus in one of the middle ears, can be found. It is chiefly in infants under the age of one year that death from convulsions of unascertainable cause occurs. Yet similar cases may be met with from time to time in children of two or three years of age. In one curious case, for example, a girl nearly three years of age was brought to a hospital for repeated convulsions. Soon after arrival a convulsion occurred which was followed by cessation of the respiration, though the pulse continued. Artificial respiration kept the child alive for over an hour. Similar cessation of respiration may occur on the termination of cases of cerebral tumours or in other cerebral affections, such as tuberculous meningitis. After death, however, in this child no abnormal condition of the brain could be found and nothing elsewhere to account for the convulsions.

### Gout.

In an interesting contribution to the *Deutsche Medizinische Wochenschrift* Dr. Cohn discusses the pathology and treatment of gout. He considers more especially the rôle played by the sodium in the formation of the urates in the gouty. Van Loghem, experimenting with rabbits, as these animals do not form uric acid, found, by injecting a certain quantity of uric acid into the back or peritoneum, that the transformation of the deposit into urates was much accelerated when sodium bicarbonate was administered to the animal at the same time. Dr. Cohn has confirmed this result, and has further tried the effect of diminishing the amount of sodium bicarbonate and increasing the potassium in the food. To effect this he either fed the rabbits on rice, which is very rich in potassium, or added potassium carbonate to the food. Under such conditions the transformation of the uric acid injected into urates was very much diminished or did not take place at all. Similar differences were obtained by injection of bromide of potassium and sodium. The author explains the deposit of urates in the cellular tissues and cartilage on the ground that these tissues are poor in potassium, and suggests that gout is rare among the Japanese on account of their large consumption of rice, and so common in England because more sodium is perhaps consumed in this country than anywhere else.

### Orthopædics and Convulsions.

Reference has already been made in these columns to Dr. Schanz's reported cases of convulsions following orthopædic operations in children, and to his view that the convulsions are caused by fatty emboli set free into the general circulation by the disturbance of the marrow during the operation. Dr. Codivilla, disagreeing with this view, suggests as an alternative hypothesis that the convulsions are caused by excessive stretching and tension of the soft parts of the limbs, in the course of the traction that is generally employed in this kind of operation, involving the nerve-trunks, and so affecting the central nervous system, especially in patients with a neuropathic taint. In the *Zentralblatt für Chirurgie* Dr. Gangele makes a further contribution to the subject. He reports two cases in which the patients, young children, underwent operation for congenital dislocation of the hip. The patients complained of a good deal of pain after the operation. In the first case four days after operation intense clonic convulsions occurred suddenly and persisted almost uninterruptedly for twenty-four hours. The patient was quite unconscious and the pupils did not react. In the second case similar convulsions occurred of a less intense nature on the second day after operation. Both patients recovered satisfactorily. Dr. Gangele favours the nerve hypothesis of Dr. Codivilla, and in support of this records the fact that in both cases the convulsions commenced immediately after the first visit of the children's parents. He urges, therefore, that after such operations the patients, especially if nervously predisposed, should be kept quite quiet and all excitement avoided. Although disagreeing with Dr. Schanz's theory of the cause of the convulsions, Dr. Codivilla and Dr. Gangele have both found the treatment recommended by him—namely, subcutaneous saline injections—very beneficial in its effects.

### Placenta Prævia.

Dr. Höhne contributes an interesting article to the *Zentralblatt für Gynækologie* upon the causal factors in the formation of placenta prævia. He accepts the general view that placenta prævia is the result of the early development of the ovum in the lower part of the uterus. Dr. Höhne is of opinion that this localisation of the ovum is largely determined by the ciliated epithelium of the matrix. In the normal state the ciliated condition of the uterine epithelium varies very much with the different physiological periods. In the premenstrual period the movements of the cilia diminish. They disappear during menstruation, and are gradually re-established afterwards. Between the periods the undulations of the cilia are apparent, but they are short, and the cilia are very fragile. In conditions of endometritis with a thickening of the mucous membrane there is a great increase in the number and in the movements of the cilia, so that there is a continuous ciliary current, and the cilia do not even disappear during menstruation. Similar phenomena are observed in conditions of menorrhagia, myoma, and prolonged metrorrhagia following miscarriage and the preclimacteric hæmorrhages. It has been recognised that these conditions favour sterility or the development of placenta prævia, but the reason assigned has been insufficient nutrition. The author points out that this can hardly be the case, as the mucous membrane is in a condition of excessive vascularisation, and the ovum is often found to develop on very poor soil, as is shown in tubular pregnancy. Dr. Höhne explains the apparent paradox on the ground that the excessive ciliary movements in this condition drive the ovum downwards, so that it is either completely expelled or develops in the lower segment of the uterus.

### Hysteria at Five Years of Age.

A case of hysteria is recorded by Dr. Virchoubsky, which is remarkable and worthy of attention on account of the early age at which the symptoms developed. The patient was a boy, aged five years, whose mother was a hysteric. He had shown no abnormal symptoms till he was by chance witness to an attack of epilepsy. From this time he began to show signs of a nervous nature. Two months later he was frightened by a dog throwing itself upon him. The child became suddenly aphonic, and at the same time developed deglutition trouble. Food even of a fluid nature was rejected from the mouth without being swallowed. The patient was admitted to hospital and a consultation was held over the case, but the physicians were unable to agree as to the diagnosis. The child was taken into the electric department to examine the pharyngeal reflex. The noise of the electric machine and the sensation caused by the introduction of the electrode into the mouth so surprised the small boy that the aphonia disappeared as if by charm. The child at once commenced to cry and talk, and immediately afterwards took the food offered him. It was evident, therefore, that the whole condition was purely functional and hysterical.

### The Treatment of Acute Pleurisy.

An interesting article on the prognosis and treatment of acute pleurisy appears in *La Clinique* from the pen of Professor Robin. After stating that the tendency of modern observers is to attribute all pleurisies of an acute type to tuberculosis, the author states that in his experience at least 84 per cent. of cases are clinically not tuberculous. For this reason he warns the general practitioner against giving an unfavourable prognosis, unless there is definite tuberculosis or evidence of hereditary predisposition. With regard to treatment, thoracentesis should be practised for left-sided effusions. The author condemns injections of sterilised air except in cases of pulmonary œdema. A large right-sided effusion does not need tapping, but can be treated medically. A milk diet should be prescribed and the patient given 1½ gr. of calomel every hour for four hours to stimulate diuresis. Next day 7½ grs. of sodium salicylate should be taken six-hourly. If the heart be normal and the effusion right-sided, 6-grain doses of an infusion of jaborandi may be used to stimulate diuresis and diaphoresis, but the drug may give rise to cardiac disturbance. Symptoms must be treated as they arise. Thus pain may necessitate an injection of morphia or of heroin, and persistent high temperature a dose of quinine. If there be little urine, Millard's mixture may be given, consisting of potassium acetate 30 grs., potassium nitrate 30 grs., oxymel of squill 1 ounce, infusion of broom 4 ounces; one tablespoonful to be taken every hour and, if necessary, continued for two or three days. As regards after-treatment, the resorption of false membranes may be stimulated by the administration of sodium arsenate and potassium iodide, while the actual cautery may be applied over the affected area if there is a diminution of vesicular breathing or a persistence of a rub. Suspected tuberculous cases should be subjected to rest and fresh-air cures, a carefully-regulated diet, including raw meat and cod-liver oil, and respiratory gymnastics.

### A New Treatment for Venereal Papillomata.

Major A. O. B. Wroughton, in the *Journal of the Royal Army Medical Corps* for June 1911, reports a case in which the whole of the mucous membrane of the foreskin was covered with papillomata so closely packed that they fitted into one another like a mosaic; they overflowed on to the skin of the penis and also on to the glans. Below the corona glandis, extending downwards three-quarters of an inch, was a thick collar of sessile warty growth which exuded the most offensive secretion. The papillomata varied in size from that of a large pea to that of a millet seed, and projected from the surface of the organ three-quarters of an inch. The end of the penis resembled in appearance and size a small cauliflower. At first local treatment by means of astringents and antiseptics was tried, but so little headway was made that it was decided to use the *x*-rays; the first exposure was for eight minutes, and the second, five days later, for ten minutes. The third exposure, again five days after the previous one, was for 18 minutes, and after this the patient was given an exposure of 15 minutes every four days until he had had seven; after a week's interval the eighth and last exposure, also of 15 minutes duration, was given. The following changes were noticed after the third exposure—the offensive secretion had quite ceased, and some of the large warts were getting hard and dry. After the fourth, most of the growths began to shrivel. After the fifth, several growths fell off, leaving quite a healthy surface beneath, and the sessile growth was shrivelling. After the sixth, half the growths had disappeared. After the seventh, there were only three small papillomata left, at the fraenum. After the eighth, and last, these three remaining growths dropped off, leaving the organ absolutely free and with not even a trace of papillomata.

### Pruritus in Chronic Nephritis.

Seeing that intense itching of the skin is often so prominent a symptom in cases in which bile is being stored in the tissues as a result of jaundice, and also in other cases in which the tissue fluids contain an excess of sugar as the result of diabetes, it would be surprising if retention of other substances besides either sugar or bile salts might not also produce itching of so widespread and intense a character as to merit the term universal pruritus. As a matter of fact, such irritation of the skin is found occasionally in an extreme degree in patients suffering from chronic Bright's disease, and the pruritus may be the first symptom that anything is wrong at all. As a rule the kidney degeneration has been present long enough in these cases to have produced some albuminuric retinitis also, and the itching is associated with cardiac hypertrophy, accentuation of the first sound at the impulse, a ringing aortic second sound, a high blood-pressure as measured instrumentally, and the passage of an abundance of pale urine, containing sometimes a trace of albumin, sometimes none at all. The diagnosis is easy enough when the possibility that chronic Bright's disease may be the cause of the pruritus is borne in mind, but hitherto the association of the two conditions has not had that stress laid upon it that it merits. The treatment of intense pruritus is by no means easy, and the intense suffering and sleeplessness that result from the symptom may be exceedingly distressing. It has been found, however, that the pruritus of chronic Bright's disease is materially relieved by means of lumbar puncture, a procedure which may be an immense boon to the patient.

### Tobacco Amblyopia.

Some details are given in the *Ophthalmoscope* of an interesting research by S. Robson into the ætiology of tobacco amblyopia. By aspirating the smoke from pipes, cigars, and cigarettes through a column of dilute blood, and then examining the latter spectroscopically, this author has demonstrated the presence of carbon monoxide in the smoke. Now, amblyopia is said especially to affect pipe smokers, and combustion appears to be particularly imperfect in pipes. Again, snuffing and chewing tobacco leaf, when indulged in alone, produce very few cases of amblyopia; and among tobacco workers, also, amblyopia is uncommon. Robson, therefore, concludes that the gaseous elements of tobacco smoke, apart from the nicotine, play an important part in tobacco poisoning, and that carbon monoxide may well have a share in the process. Of the alkaloidal constituents of tobacco, he attaches most importance to nicotine, both for its general effects on the system and for this special degenerative lesion of the eye. He also admits that the importance of the pyridine bases—of which pyridine itself is the principal—has been under-estimated. The question to what extent nicotine is transformed into these bases during the combustion of the tobacco does not appear to be definitely settled.

### Exophthalmic Goitre in Men.

This disease is so much commoner in women that it is often regarded as *par excellence* a feminine complaint. Exact collation shows, however, that some 18 per cent. of patients are men; Pic and Bonnamour have collected a hundred and nine cases in men, and they analyse the special characteristics of the condition as it occurs in the male sex. Tachycardia, as with women, is one of the most constant signs; and tremor is also seldom absent. Palpitation, anorexia, and diarrhœic attacks are all common. Enlarged thyroid is not universally present, as is the case in women, thus constituting the paradox of "exophthalmic goitre" with no goitre to be found. Wasting is usually well marked; exophthalmos is, of course, common, but not universal. The nervous symptoms are frequently very severe: irritability, excitement, and unstable equilibrium all round are often met with. The slightest noise or other disturbance may provoke an explosion of wrath, and control is very markedly lessened. Delusions of persecution and hallucinations are also encountered, and complete mental breakdown may follow. The prognosis is very bad, much worse than in women; the patients go rapidly downhill, and pneumonia is a frequent terminal phenomenon. The authors have little to suggest beyond the ordinary treatment, except to recommend surgical intervention.

### Digital Absorption in Raynaud's Disease.

Professor Wardrop Griffith contributes to the *Medical Chronicle* some interesting observations on a patient whom he has attended for Raynaud's disease since 1889. Photographs, sketches, and clinical memoranda all show that there has been progressive shortening of the digits, which are now greatly reduced in size: indeed, the terminal phalanges are almost gone, and the other bones are considerably thinned and shortened. A series of excellent *x*-ray photographs show beyond dispute that this is the case, and Professor Griffith emphasises the fact that at no time has there ever been any loss of substance by gangrene; there is still some indication of a nail on each of the fingers. The shortening must, then, be due to interstitial absorption. Not only are the phalanges shortened; they are even more markedly thinned, and in some cases bent and distorted. There is complete bony ankylosis of some joints. The contentions put forward by Dr. Griffith are certainly borne out by the photographs reproduced; but we seem to be as far as ever from any satisfactory theory of the causation of these deformities and from any successful method of treatment.

